# Risk tools for predicting long-term sequelae based on symptom profiles after known and undetected SARS-CoV-2 infections in the population

**DOI:** 10.1007/s10654-025-01223-y

**Published:** 2025-05-19

**Authors:** Rieke Baumkötter, Simge Yilmaz, Julian Chalabi, Vincent ten Cate, Ayesha Syed Mamoor Alam, Sepehr Golriz Khatami, Daniela Zahn, Nora Hettich-Damm, Jürgen H. Prochaska, Irene Schmidtmann, Kristin Lehnert, Anke Steinmetz, Marcus Dörr, Norbert Pfeiffer, Thomas Münzel, Karl J. Lackner, Manfred E. Beutel, Philipp S. Wild

**Affiliations:** 1 Preventive Cardiology and Preventive Medicine, Center for Cardiology, University Medical Center of the Johannes Gutenberg University Mainz, Langenbeckstr. 1, 55131 Mainz, Germany; 2 Partner Site Rhine Main, German Center for Cardiovascular Research (DZHK), University Medical Center of the Johannes Gutenberg University Mainz, Mainz, Germany; 3 Center for Thrombosis and Hemostasis (CTH), University Medical Center of the Johannes Gutenberg University Mainz, Langenbeckstr. 1, 55131 Mainz, Germany; 4 Health Sciences, Hochschule Fulda University of Applied Sciences, Leipziger Str. 123, 36037 Fulda, Germany; 5 Department of Psychosomatic Medicine, University Medical Center of the Johannes Gutenberg University Mainz, Untere Zahlbacher Str.8, 55131 Mainz, Germany; 6 Institute of Medical Biometry, Epidemiology and Informatics, University Medical Center of the Johannes Gutenberg University Mainz, Rhabanusstraße 3, Tower A, 55131 Mainz, Germany; 7 Department of Internal Medicine B, University Medicine Greifswald, Fleischmannstraße 8, 17475 Greifswald, Germany; 8 German Center for Cardiovascular Research (DZHK), Partner Site Greifswald, Greifswald, Germany; 9 Physical and Rehabilitation Medicine, Department of Orthopaedics,Trauma and Rehabilitation Medicine, University Medicine Greifswald, Ferdinand-Sauerbruch-Straße, 17475 Greifswald, Germany; 10 Department of Ophthalmology, University Medical Center of the Johannes Gutenberg University Mainz, Langenbeckstr. 1, 55131 Mainz, Germany; 11 Cardiology I, Center for Cardiology, University Medical Center of the Johannes Gutenberg University Mainz, Langenbeckstr. 1, 55131 Mainz, Germany; 12 Institute of Clinical Chemistry and Laboratory Medicine, University Medical Center of the Johannes Gutenberg University Mainz, Langenbeckstr. 1, 55131 Mainz, Germany; 13 Institute of Molecular Biology (IMB), Ackermannweg 4, 55128 Mainz, Germany

**Keywords:** Post-COVID syndrome, SARS-CoV-2, Machine learning, Risk assessment, Diagnostic tools, Population-based cohort study

## Abstract

The aim was to determine the profile of long-term symptoms after known and undetected SARS-CoV-2 infections and to generate tools for risk and diagnostic assessment of Post-COVID syndrome (PCS). In the population-based Gutenberg COVID-19 Study (*N* = 10,250), sequential, systematic screening for SARS-CoV-2 was performed in 2020/2021. Individuals received a standardized interview on newly occurred or worsened symptoms since the infection or the pandemic. Robust Poisson regression models were fit to compare the frequency of symptoms between groups. Two scores were developed using machine learning techniques and prospectively validated in an independent cohort. Among *n* = 942 individuals, prevalence of long-term symptoms was 36.4% among individuals with known SARS-CoV-2 infection, 25.0% in those unknowingly infected, and 28.1% among the controls. Individuals with known infection more often reported smell (Prevalence ratio [PR] = 13.66 [95% confidence interval 4.99;37.41]) and taste disturbances (PR = 5.57 [2.62;11.81]), forgetfulness (PR = 2.88 [1.55;5.35]), concentration difficulties (PR = 2.83 [1.55;5.16], trouble with balance (PR = 2.74 [1.18;6.35]), and dyspnea (PR = 2.22 [1.18;4.19]) than controls. The risk score for predicting long-term sequelae based on symptoms during the acute infection had a cross-validated AUC of 0.74 and 0.72 when applied in an independent cohort (*N* = 6,570). The diagnostic score providing a probability of the presence of PCS had a cross-validated AUC of 0.66 and of 0.64 in the validation cohort (*N* = 3,176). Individuals with and without SARS-COV-2 infection reported persistent symptoms, but symptoms attributable to PCS were identified. The data-driven scores may help guide further diagnostic decisions in the initial management of PCS.

**Supplementary Information:**

The online version contains supplementary material available at 10.1007/s10654-025-01223-y.

## Introduction

Post-COVID syndrome (PCS) is characterized by heterogeneous long-term sequelae following infection with severe acute respiratory syndrome coronavirus 2 (SARS-CoV-2) and remains poorly understood [1–3]. Persistent symptoms after coronavirus disease 19 (COVID-19) had long been inconsistently defined. The late introduction of a standard nomenclature of PCS and resulting differences in methodological approaches of studies complicate the estimation of its prevalence in the general population: a meta-analysis of 41 studies found a prevalence of PCS (defined as symptoms persisting for at least 28 days after SARS-CoV-2 infection) ranging from 9–81% [4]. PCS has been associated with an enormous range of symptoms without clinical pattern, suggesting it to be a multi-organ disease [5–7]. Due to these multifaceted symptoms and the lack of established diagnostic biomarkers, PCS remains a diagnosis of exclusion requiring extensive diagnostic efforts [5, 8]. 

Earlier studies on PCS have frequently examined only individuals with a history of SARS-CoV-2 infection, without including a comparison group of non-infected individuals [7, 9–12]. This has prevented the differentiation between long-term symptoms associated with SARS-CoV-2 and symptoms caused by other diseases. Much of the evidence on PCS is also based on cohorts of hospitalized patients [13–17] who have expected late effects of post-critical illness (i.e., post-intensive care syndrome). Given that most individuals with SARS-CoV-2 infections had a mild course that did not require inpatient medical treatment, there is an urgent need to systematically investigate long-term symptoms in a population-based sample that captures the full range of SARS-CoV-2 infection severity.

The aims of this study were (i) to determine the profile of long-term symptoms among individuals with a history of known and undetected SARS-CoV-2 infection compared with non-infected controls in a population-representative cohort study, (ii) to develop data-driven scores for the risk assessment and the diagnostic assessment of PCS, and (iii) to prospectively validate them in an independent population-based cohort.

## Methods

### Study design

Data from the Gutenberg COVID-19 Study (GCS, *N* = 10,250 participants), a prospective population-based cohort study in Germany, were analyzed. Details about the study design and data collection are described elsewhere [18]. Briefly, the study sample was randomly drawn by regional registration offices with stratification by sex assigned at birth (male/female), age (25–88 years), and place of residence (City of Mainz/District of Mainz-Bingen). Participants had to be able to visit the study center and to understand the German language sufficiently. The sample consisted of 8,121 individuals aged 45–88 years participating in the population-based Gutenberg Health Study [19] corresponding to 79% of the total cohort, and 2,129 newly recruited individuals from a random sample aged 25–44 years. The study program consisted of a baseline examination (October 2020 to April 2021) and a follow-up investigation (March to June 2021) at a dedicated study center, a computer-assisted telephone interview (CATI, August 2021 to January 2022), and a questionnaire-based long-term follow-up (May to November 2022). The granular data were derived from biomaterial samples, computer-assisted face-to-face and telephone interviews, and questionnaires. The prevalence of comorbidities was assessed on the basis of self-reported data and, for participants in the Gutenberg Health Study, by medical-technical examinations during the study and medical records. Socioeconomic status (SES) was operationalized using the socioeconomic index of Lampert & Kroll, which considers education, occupation, and income [20]. The index values range from 3 (lowest SES) to 21 (highest SES).

### Identification of SARS-CoV-2 infections

SARS-CoV-2 infections were screened in a multimodal manner. Individuals were deemed infected if either quantitative reverse transcription polymerase chain reaction (RT-qPCR) or antibody measurements were positive, or based on self-reports from computer-assisted personal interviews and weekly smartphone app-based reports [18]. RT-qPCR was used to detect acute infections using the Light Mix SarbecoV E-gene (plus EAV control) and RdRP-gene (TIB Molbiol, Germany) [21]. EDTA plasma samples were analyzed for antibodies to the SARS-CoV-2 nucleocapsid protein using two immunoassays (Architect SARS-CoV-2 IgG, Abbott, Germany and Elecsys Anti-SARS-CoV-2 Pan-Ig, Roche, Germany). SARS-CoV-2 infections were considered undetected if RT-qPCR or antibody measurements were positive without self-report of an infection. Individuals were informed about their test results. Information on sample storage, preprocessing, and measurements are provided in the Supplementary Appendix.

### Assessment and definition of acute and long-term symptoms

Individuals with a history of SARS-CoV-2 infection were interviewed by CATI about the symptoms of the acute infection and sequelae between August 2021 and January 2022, i.e., after the baseline and the first follow-up investigation. Participants were asked about 61 symptoms according to the WHO Case Report Form for Post-COVID condition (Post COVID-19 CRF) [22]. For each symptom, individuals with a known history of SARS-CoV-2 were asked about the duration (0–3 months, > 3–6 months, or ≥ 6 months) after infection (Supplemental Table 1). Individuals with a history of an undetected infection were asked about the duration of symptoms since the onset of the pandemic in Germany (February 1st, 2020), as the date of infection was unknown. Both groups were asked about severity and whether the complaints had been present in that frequency and intensity before infection or pandemic, respectively. The time of 0–3 months was considered the “acute phase of SARS-CoV-2 infection”, 3–6 months the “post-acute phase”, and symptoms persisting for at least 6 months were defined as long-term symptoms (“post-COVID phase”). Individuals free of SARS-CoV-2 infection, i.e., negative in both antibody assays, negative in RT-qPCR, and without self-reported positive SARS-CoV-2 tests, were defined as the control group. Controls were selected in a 1:1 ratio to individuals with a history of SARS-CoV-2 infection, with age and sex matching at group-level. They were screened identically to persons with a history of undetected SARS-CoV-2 infection.

In all groups, only symptoms that were new-onset or worsened since the infection or onset of the pandemic were considered in analyses.

### Statistical analysis

The analysis sample included all individuals with a known or undetected history of SARS-CoV-2 infection and age-sex-matched controls free of SARS-CoV-2 who received the CATI with comprehensive assessment of acute and long-term symptoms. Continuous data were summarized using median and interquartile range (IQR) and categorical variables were described by absolute and relative frequencies. Poisson regression models with robust standard errors were fit to compare the prevalence of symptoms in individuals with history of a known or undetected SARS-CoV-2 infection against that in individuals without a history of SARS-CoV-2. Models were adjusted for age, sex, and SES. Generalized additive models were used to predict the probability of sequelae after 3 and 6 months (yes vs. no) based on the number of symptoms during the acute infection with SARS-CoV-2 among individuals with a history of a known infection. Robust Poisson regression with adjustment for age, sex, and SES was used to identify clinical risk factors of sequelae.

Two scores were developed using machine learning techniques. The “GCS Post-COVID Risk Score” is a prognostic score used to predict the probability of having long-term sequelae at least 6 months after SARS-CoV-2 infection based on symptoms present during the acute infection phase. Both individuals with a history of known and undetected SARS-CoV-2 infection were included. Regularized regression (alpha = 0.1) selected symptoms during the acute phase of SARS-CoV-2. The second score, the “GCS Post-COVID Diagnostic Score”, provides a probability describing how likely it is that reported long-term symptoms are related to PCS. Long-term symptoms persisting for at least 6 months were selected by regularized regression that differentiate between being seropositive or having a positive PCR or antigen test result. Individuals with a history of known and undetected SARS-CoV-2 infection as well as the control group were included. The derived penalized estimates were used as weights for the scores. The penalization parameter λ for both models was identified by minimizing the binomial deviance in the holdout sample in 10-fold cross-validation. For both scores, the selected symptoms were ranked according to their predictive strength by lambda ratio, a scale-invariant measure of the predictive robustness of each symptom. The lambda ratio is defined as the ratio between the value of λ at which a given variable’s coefficient estimate was first shrunk to zero to the optimal λ selected by cross-validation. A cut-off for the scores was a priori chosen to achieve a sensitivity of 95% with the highest possible specificity to ensure that individuals not identified as positive by the scores can safely be ruled out. Positive and negative predicted values were calculated using the Bayes formula to account for the prevalence.

All analyses were of exploratory nature, with p-values (*P*) considered as a continuous measure of statistical evidence. Statistical analyses were performed using the statistical software package R, version 4.2.1 (R Foundation for Statistical Computing, Vienna, Austria).

### Independent population-based cohort to prospectively validate the post-COVID scores

SentiSurv RLP, a surveillance and early warning system for SARS-CoV-2 infections in Rhineland-Palatinate, Germany [23, 24], was used as an external and independent validation cohort for both scores. It is a prospective, population-based setting in which participants conducted SARS-CoV-2 rapid antigen tests weekly and transmitted the results along with additional data via a smartphone application. Information about the symptoms from the developed scores were used to prospectively collect the needed data in SentiSurv RLP.

## Results

The analysis sample comprised 942 individuals from the total sample of 10,250 individuals, including 272 with a known history of SARS-CoV-2, 200 with a history of an undetected infection, and 470 persons without a history of SARS-CoV-2 (Table 1). The sex distribution was similar across groups (proportion of women: in known infections 50.0%, undetected infections 47.0%, controls 48.5%). Individuals with a history of undetected SARS-CoV-2 infection were the oldest (58.1 [43.7/69.6] years) compared with individuals with a known infection (51.5 [40.3/61.2] years) and individuals free of SARS-CoV-2 (54.6 [41.3/65.6] years). The minority was hospitalized due to COVID-19 (5.1%) or received outpatient treatment (3.3%). The median time between the first positive test result and follow-up via CATI was approximately 9 months. In June 2021, 43.4% of the analysis sample were vaccinated against SARS-CoV-2 and 9.7% of individuals with a known history of SARS-CoV-2 infection were vaccinated at the time of infection.

**Table 1:** Characteristics of cohort sample stratified by infection status

	History of known SARS-CoV-2 infection *N* = 272	History of undetected SARS-CoV-2 infection *N* = 200	Control group without SARS-CoV-2 infection *N* = 470
*Sociodemographic data*			
Sex (women), [%] (n)	50.0 (136)	47.0 (94)	48.5 (228)
Age [years] (IQR)	51.5 (40.3/61.2)	58.1 (43.7/69.6)	54.6 (41.3/65.6)
Socioeconomic status (IQR)	16.0 (12.0/19.0)	14.0 (12.0/18.0)	15.0 (12.0/18.0)
*Traditional cardiovascular risk factors*,* [%] (n)*			
Arterial hypertension	42.6 (116)	48.2 (96)	46.6 (219)
Diabetes mellitus	9.9 (27)	6.5 (13)	6.8 (32)
Dyslipidemia	29.2 (79)	36.7 (73)	35.8 (168)
Obesity	23.2 (63)	20.5 (41)	20.9 (98)
Smoking (current)	11.8 (32)	17.0 (34)	17.7 (83)
*Clinical profile*,* [%] (n)*			
Anxiety	14.6 (39)	3.3 (6)	4.9 (23)
Autoimmune disease	7.4 (20)	6.0 (12)	9.4 (44)
Cardiovascular disease	8.9 (24)	17.6 (35)	10.6 (50)
Atrial fibrillation	1.9 (5)	3.1 (6)	3.0 (14)
Coronary artery disease	3.0 (8)	6.5 (13)	3.8 (18)
Heart failure	1.1 (3)	4.0 (8)	1.9 (9)
Hx. of myocardial infarction	2.2 (6)	4.5 (9)	2.6 (12)
Hx. of stroke	0.4 (1)	2.5 (5)	1.9 (9)
Peripheral artery disease	3.7 (10)	6.6 (13)	2.3 (11)
Chronic kidney disease	3.7 (10)	3.5 (7)	3.6 (17)
Chronic liver disease	2.6 (7)	1.5 (3)	2.3 (11)
Chronic obstructive pulmonary disease	3.3 (9)	3.5 (7)	6.0 (28)
Depression	7.8 (21)	3.9 (7)	5.2 (24)
Hx. of cancer	9.2 (25)	12.5 (25)	13.2 (62)
Hx. of venous thromboembolism	4.8 (13)	6.0 (12)	6.0 (28)
*Number of symptoms during 0–3 months after SARS-CoV-2 infection*,* [%] (n)*			
0 symptoms	8.1 (22)	0.5 (1)	1.3 (6)
1–5 symptoms	5.1 (14)	28.3 (53)	37.7 (177)
6–10 symptoms	22.1 (60)	48.7 (91	39.8 (187)
11–15 symptoms	19.1 (52)	4.3 (8)	4.5 (21)
16–20 symptoms	10.7 (29)	2.7 (5)	1.9 (9)
≥20 symptoms	34.9 (95)	15.5 (29)	14.9 (70)
*SARS-CoV-2 related characteristics*			
SARS-CoV-2 vaccination*, [%] (n)	34.9 (88)	41.2 (75)	48.7 (229)
Vaccination at time of infection, [%] (n)	9.7 (7)	unknown	n.a.
Inpatient treatment, [%] (n)	5.1 (14)	0.1 (1)	n.a.
Outpatient treatment, [%] (n)	3.3 (9)	0 (0)	n.a.
Time since first positive test [months] (IQR)	8.55 (6.84/10.30)	n.a.	n.a.

Presented are medians with interquartile ranges (IQR) or absolute and relative frequencies

* All SARS-CoV-2 vaccinations during the course of the study

Hx, history; n.a., not applicable

### Reported symptoms in infected and uninfected individuals

**Fig. 1:**
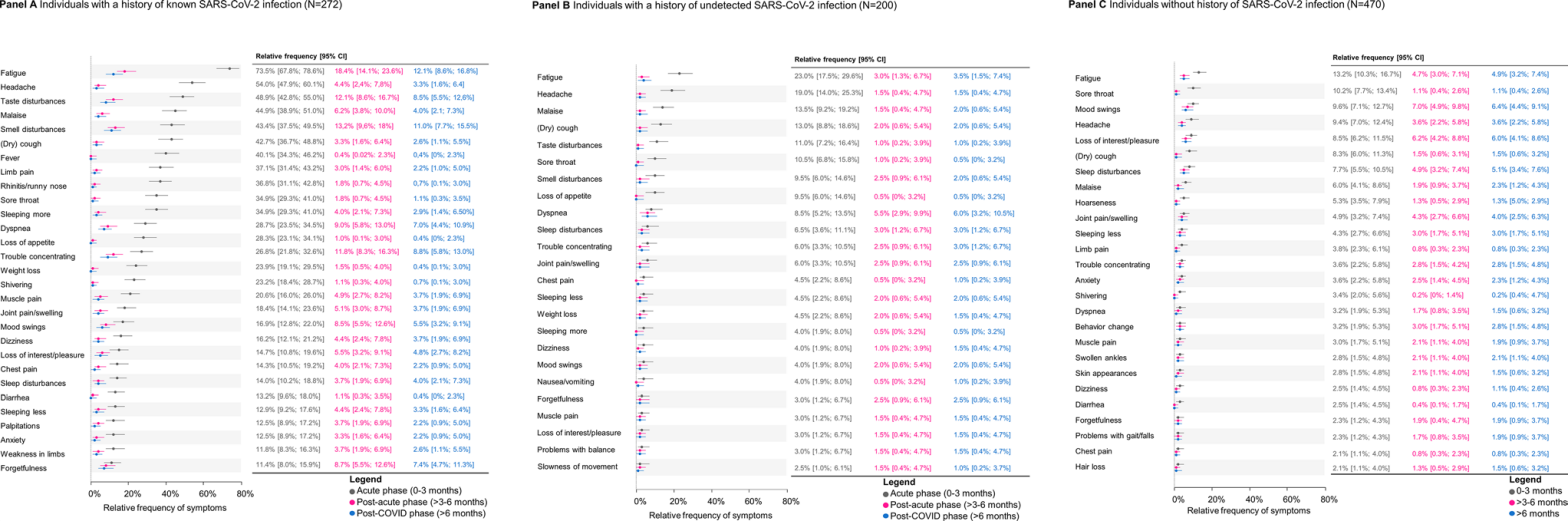
Reported symptoms during 0–3 months (acute phase), > 3–6 months (post-acute phase), and > 6 months (post-COVID phase) stratified by infection status Symptoms are shown stratified for individuals with a known history of SARS-CoV-2 infection (Panel A), individuals with a history of undetected SARS-CoV-2 infection (Panel B) and individuals without history of SARS-CoV-2 infection (Panel C). Symptoms are ranked according to their relative frequency during 0–3 months. In Panel A, all symptoms with a prevalence of at least 10% in the acute phase are shown and in Panel B and C, a threshold of 2% was used

The prevalence of symptoms during the acute phase (0–3 months), the post-acute phase (> 3–6 months, and the post-COVID phase (> 6 months) are shown in Fig. 1, stratified by infection status. In individuals knowingly infected with SARS-CoV-2, forgetfulness was observed to be relatively stable over time, while the relative frequency of most symptoms decreased sharply from the acute to the post-COVID phase (Fig. 1, **Panel A**). Fever, rhinitis, loss of appetite, weight loss, shivering, and diarrhea were common only during the acute phase. In contrast, fatigue, smell and taste disturbances, dyspnea, difficulty concentrating, and forgetfulness were still present in the post-COVID phase. Among individuals with a history of undetected SARS-CoV-2 infection, the prevalence of symptoms was generally lower than among those with a known history of SARS-CoV-2. Reported long-term symptoms were comparable to those with a history of known infection, although less prevalent (Fig. 1, **Panel B**). Among individuals without history of SARS-CoV-2 infection, fatigue was one of the most mentioned symptoms persisting for at least 6 months, followed by mood swings, loss of interest/pleasure, and sleep disturbances (Fig. 1, **Panel C**).

Age and sex differences were investigated in the ten most reported long-term complaints among individuals with a history of known or undetected SARS-CoV-2 infection (Supplemental Fig. 1). The age-dependency of long-term smell and taste disturbances differed by sex, with younger men and older women experiencing altered smell and taste, respectively (smell disturbances: *P*_age*sex_=0.059, *P*_age_=0.017, *P*_sex_=0.070; taste disturbances: *P*_age*sex_=0.030, *P*_age_=0.070, *P*_sex_=0.048). No interaction between age and sex was found for the remaining symptoms.

### Prevalence of symptomatic individuals and symptom burden over time

**Fig. 2:**
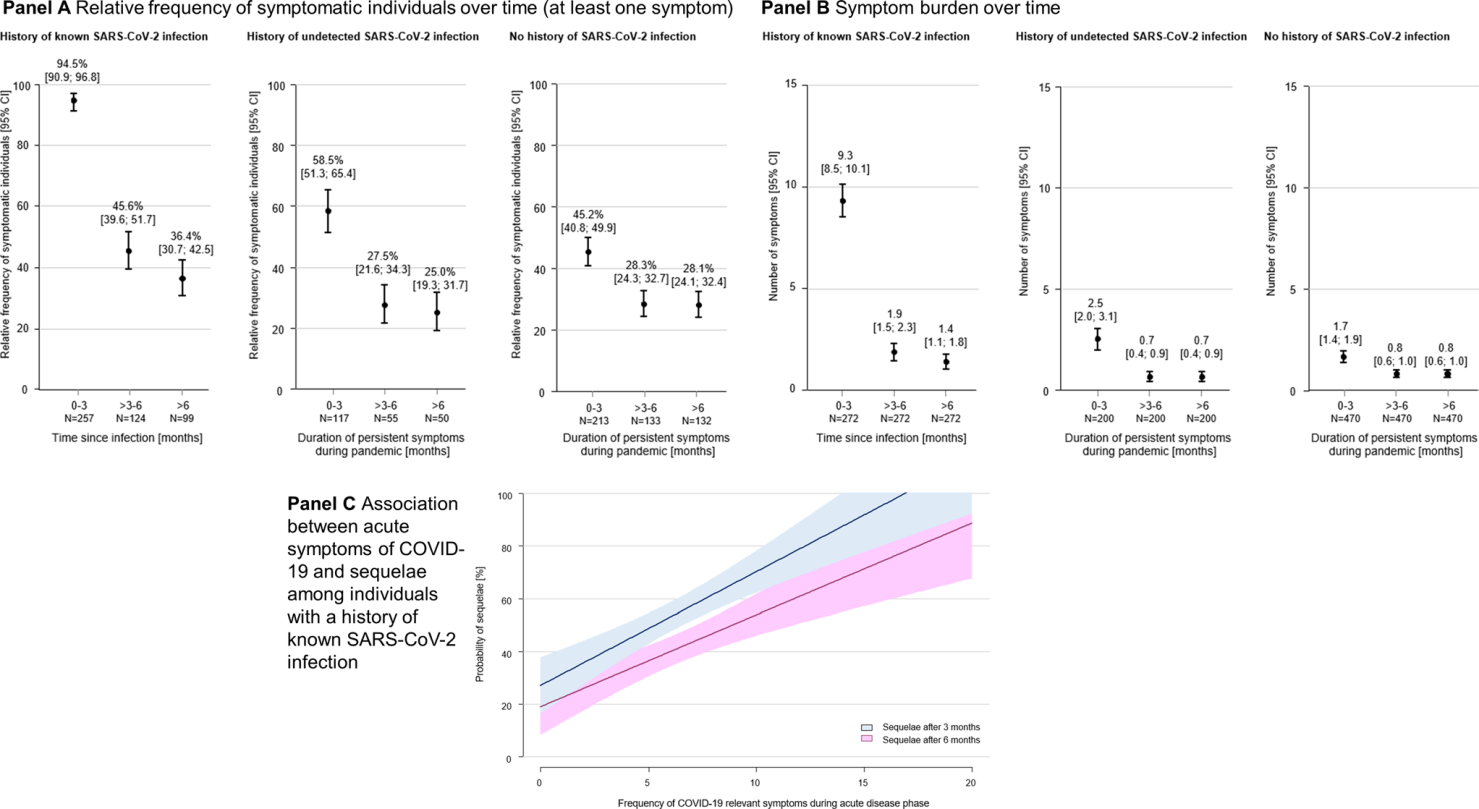
Prevalence of symptomatic individuals over time and the association between symptom burden and sequelae. Relative frequency of symptomatic individuals over time (at least one symptom, Panel A) and symptom burden over time (Panel B). For individuals with a known history of SARS-CoV-2, newly occurred or worsened symptoms since the SARS-CoV-2 infection is shown. For individuals with a history of undetected SARS-CoV-2 or without history of SARS-CoV-2 infections, newly occurred or worsened symptoms since the onset of the pandemic (February 2020) were used. Bars represent 95% confidence intervals (CI). The N refers to the number of individuals who reported symptoms. Panel C displays the association between COVID-19-relevant symptoms during the acute phase and the probability of having sequelae among individuals with a history of known SARS-CoV-2 infection using generalized additive models with smoothness estimation. COVID-19-relevant symptoms include fever, cough, sore throat/throat scratching, rhinitis/runny nose, headache, dyspnea/shortness of breath, pain on breathing, whistling/wheezing breathing, chest pain, palpitations, joint pain/swelling, limb pain, nausea/vomiting, seizures, red-purple discoloration on toes, body/face paralysis, fatigue, shivering, dizziness, weight loss, weakness in limbs, problems with gait/falls, diarrhea, smell or taste disturbances

The proportion of symptomatic individuals decreased in both groups with a history of known and undetected SARS-CoV-2 infection over time (Fig. 2, **Panel A**). The prevalence of individuals with at least one persisting symptom for a minimum of six months was 36.4% (95% confidence interval [CI] 30.7%; 42.5%) among individuals with a known history of SARS-CoV-2 and 25.0% (19.3%; 31.7%) in individuals with a history of undetected infection. Regarding individuals without history of SARS-CoV-2 infection, 28.1% (24.1%; 32.4%) reported persisting symptoms. Mean number of symptoms decreased among individuals with a history of known SARS-CoV-2 infection over time, while persons with a history of undetected infection and the uninfected had stable symptom burden after three months (Fig. 2, **Panel B**). In individuals with a known history of SARS-CoV-2 infection who were vaccinated at the time of infection, 2 out of 7 individuals (~ 30%) reported the persistence of symptoms after six months, in contrast to ~ 45% (33%; 57%) of individuals who had not received vaccination at the time of infection. Among individuals with a known history of SARS-CoV-2, the prevalence of sequelae was associated with the number of COVID-19-relevant symptoms during the acute infection. The more symptoms occurred in the acute phase, the higher the likelihood of being affected by sequelae that lasted at least 3 months (estimate = 5.37, *P* < 0.0001) or 6 months (estimate = 3.64, *P* < 0.0001, Fig. 2, **Panel C**).

### Profile of long-term symptoms

**Fig. 3:**
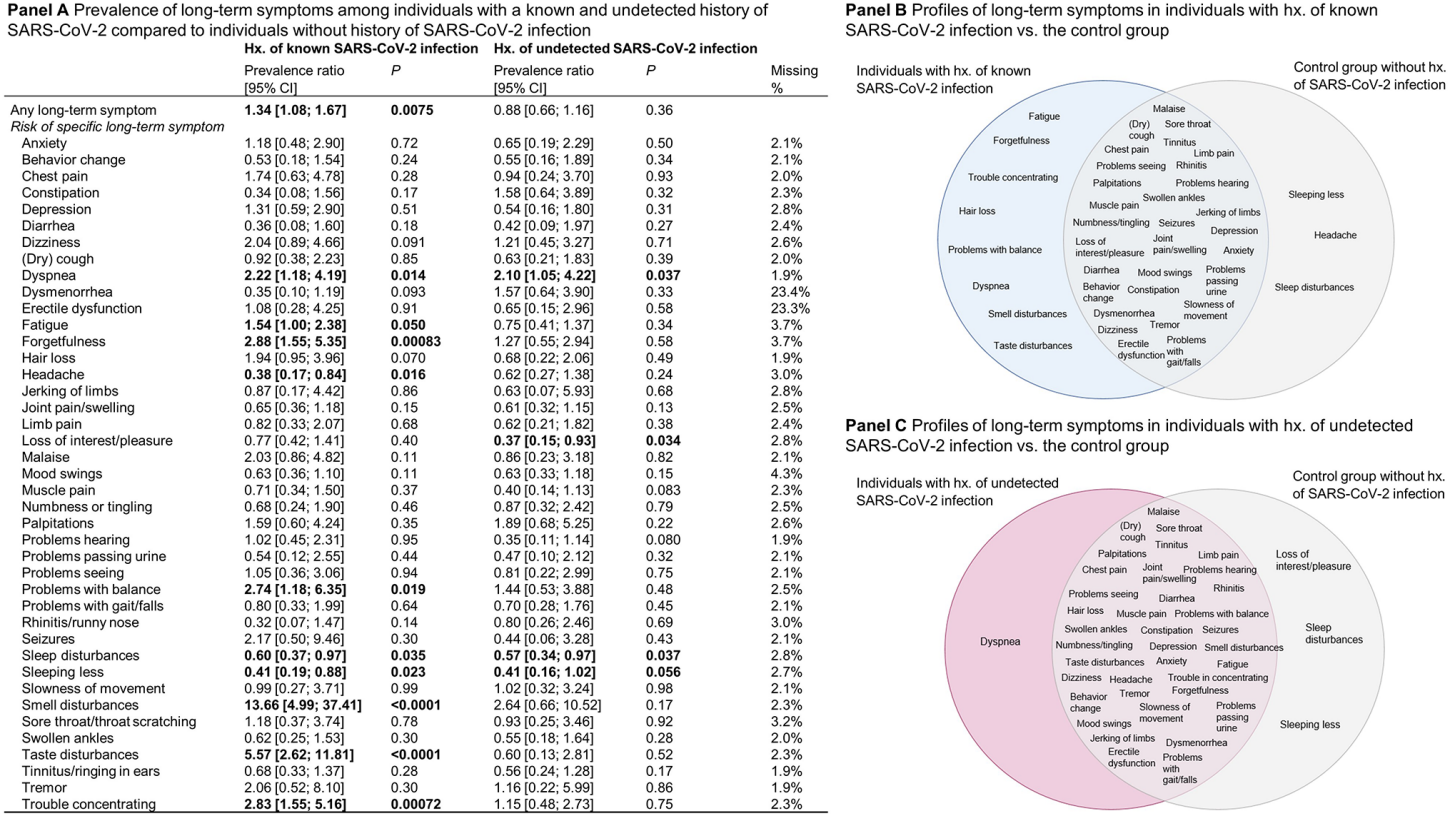
Differences in symptoms during post-COVID phase (> 6 months). Poisson regression models with robust standard errors adjusted for age, sex, and socioeconomic status (Panel A) and Venn-diagrams showing the derived symptom profiles (Panel B and Panel C). Symptoms that could not be analyzed due to low prevalence in one of the two groups being compared (*n* ≤ 2): fever, shivering, hoarseness, whistling breathing/wheezing breathing, conjunctivitis, confusion/consciousness disorders, sleeping more, fainting, stiffness of muscles, weakness in limbs, abdominal pain, nausea/vomiting, loss of appetite, weight loss, appearances, problems swallowing, hallucinations, (purple/pink/bluish) lumpy lesions on toes (COVID toes), can‘t move and/or feel one side of body or face. CI, confidence interval; hx, history of

The prevalence of any long-term symptom persisting for at least 6 months was higher in individuals with a known history of SARS-CoV-2 infection compared to persons without SARS-CoV-2 (PR = 1.34 [95% CI 1.08; 1.67], Fig. 3, **Panel A**). Specifically, individuals with a history of known infection had a higher prevalence of dyspnea (PR = 2.22 [1.18; 4.19]), fatigue (PR = 1.54 [1.00; 2.38]), forgetfulness (PR = 2.88 [1.55; 5.35]), problems with balance (PR = 2.74 [1.18; 6.35]), smell disturbances (PR = 13.66 [4.99; 37.41]), and trouble concentrating (PR = 2.83 [1.55; 5.16]). Headache, sleep disturbances, sleeping less, and loss of interest or pleasure were reported more commonly by individuals without history of SARS-CoV-2. The derived symptom profiles of the groups are shown in Fig. 3, **Panel B**.

Dysmenorrhea (PR = 0.22 [0.06; 0.82]), fatigue (PR = 2.14 [1.15; 4.00]), smell disturbances (PR = 4.95 [1.81; 13.59]), and trouble concentrating (PR = 2.40 [1.09; 5.29]) were found to be symptoms that discriminate between individuals with a history of a known and a history of an undetected SARS-CoV-2 infection (Supplemental Table 2).

### Clinical risk factors for sequelae

**Fig. 4:**
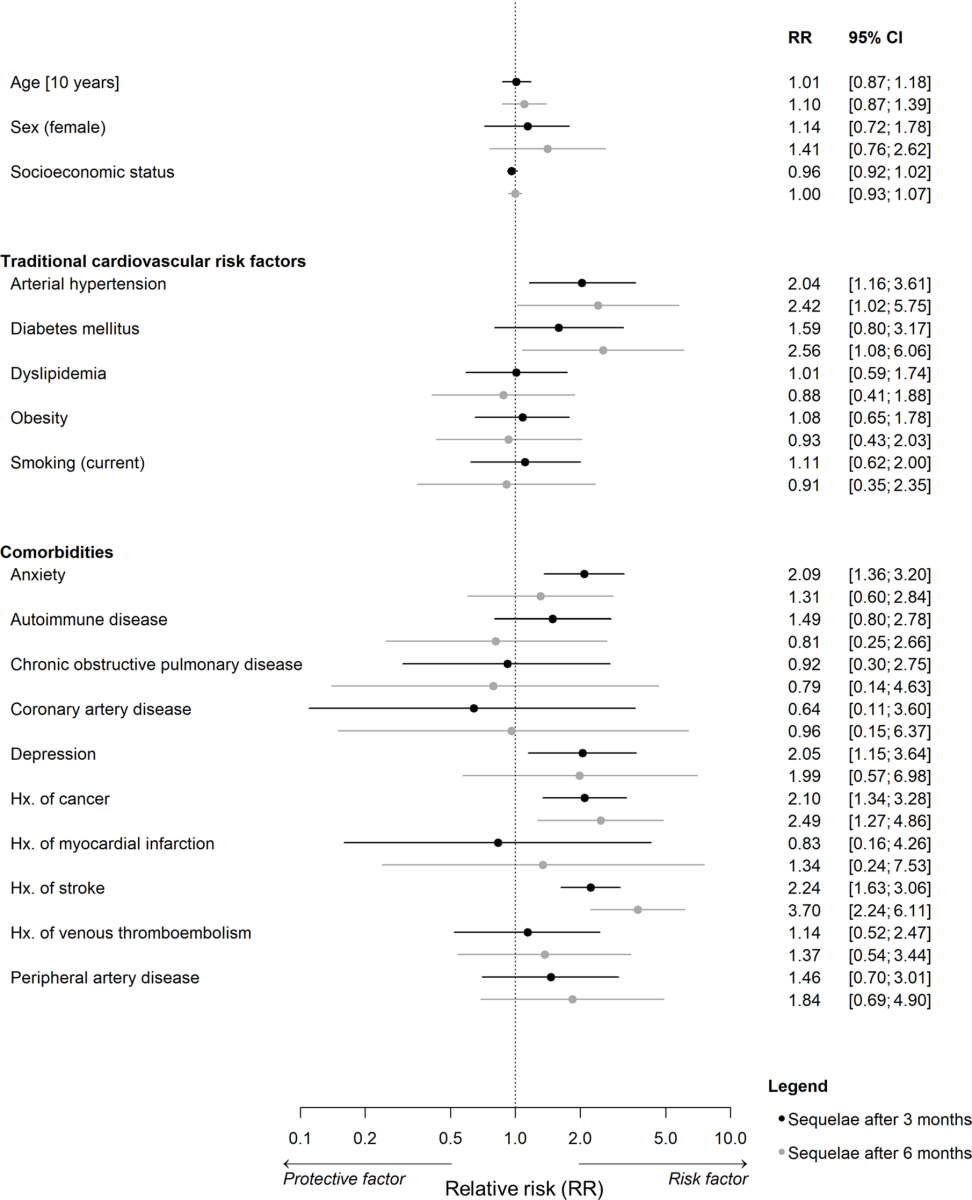
Clinical risk factors for sequelae. Poisson regression with robust standard errors adjusted for age, sex, and socioeconomic status among individuals with a known and undetected history of SARS-CoV-2 infection. Clinical data at baseline were predictors and SARS-CoV-2 infections occurring between baseline and follow-up were used for outcome (*N* = 106). Atrial fibrillation, chronic kidney disease, and chronic liver disease were excluded due to small sample size. SES, socioeconomic status; RR, risk ratio; CI, confidence interval

Among individuals with known and undetected infection, a higher risk for long-term sequelae was observed for individuals with diabetes mellitus (RR = 2.56 [1.08; 6.06], Fig. 4). Depression (RR = 2.05 [1.15; 3.64]) and anxiety (RR = 2.09 [1.36; 3.20]) were identified as risk factors for sequelae after 3 months. Persons with arterial hypertension, a history of cancer and of stroke had an increased risk for both, sequelae after 3 months and sequelae after 6 months.

### Data-driven scores for risk and diagnostic assessment for post-COVID

**Fig. 5:**
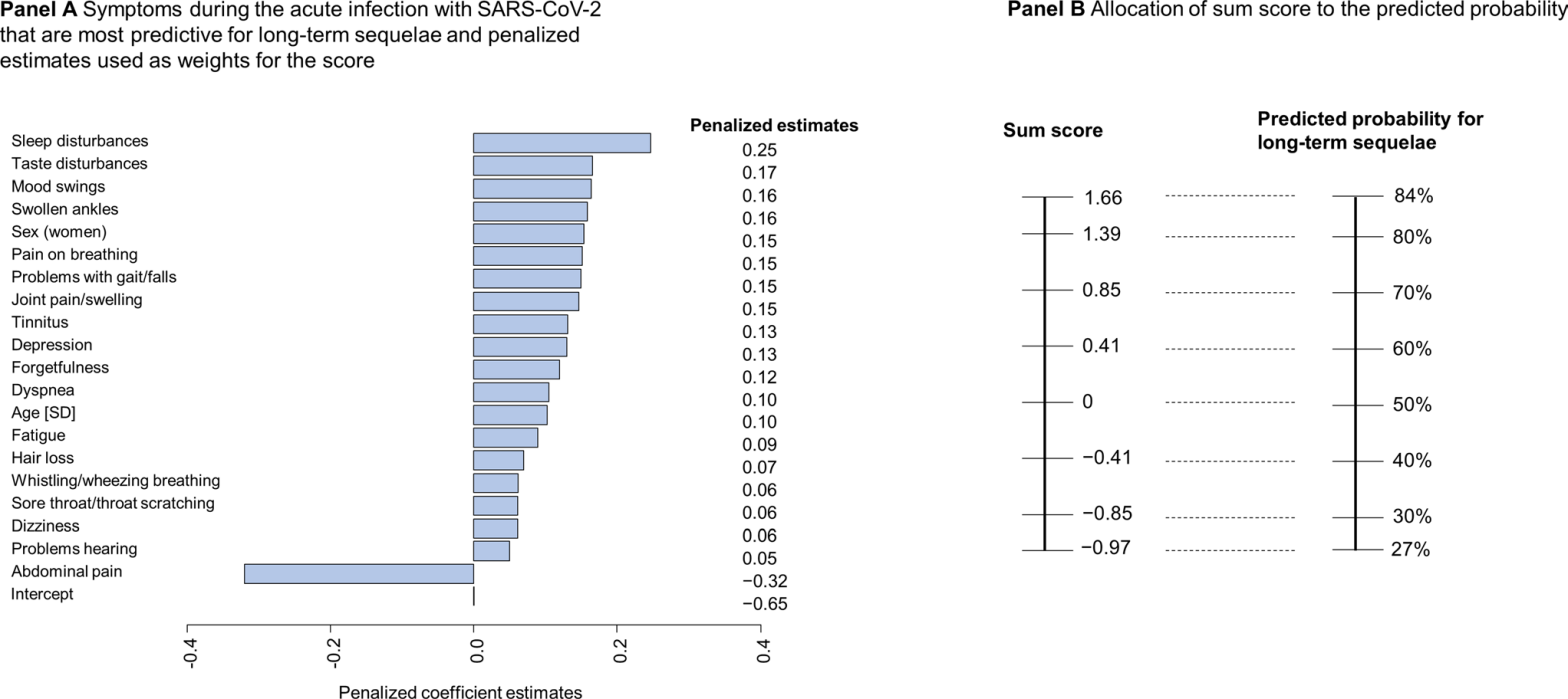
Development of the GCS Post-COVID Risk Score. Panel A shows the penalized estimates of the selected symptoms with regularized regression (adjustment for sex and age, AUC = 0.79, 10-fold cross-validation AUC = 0.74, minimal lambda = 0.093, *N* = 406, number of symptoms = 53, selected symptoms = 18). Panel B presents the allocation of the sum score to the predicted probability

With regard to the GCS Post-COVID Risk Score, 18 symptoms were selected by machine learning which are most predictive for symptoms during the post-COVID phase in addition to age and sex (Fig. 5, **Panel A**). All symptoms but abdominal pain had a positive correlation with long-term sequelae. By summing the weights for each symptom present, the sum score is obtained, which can be converted to a predicted probability of the presence of long-term symptoms at least 6 months after infection (Fig. 5, **Panel B**). The derivation model had an AUC of 0.79 (cross-validated AUC = 0.74) with a sensitivity of 94% and a specificity of 20% (positive predicted value [PPV] = 35%, negative predicted value [NPV] = 87%, **Supplemental Fig. 2**).

**Fig. 6:**
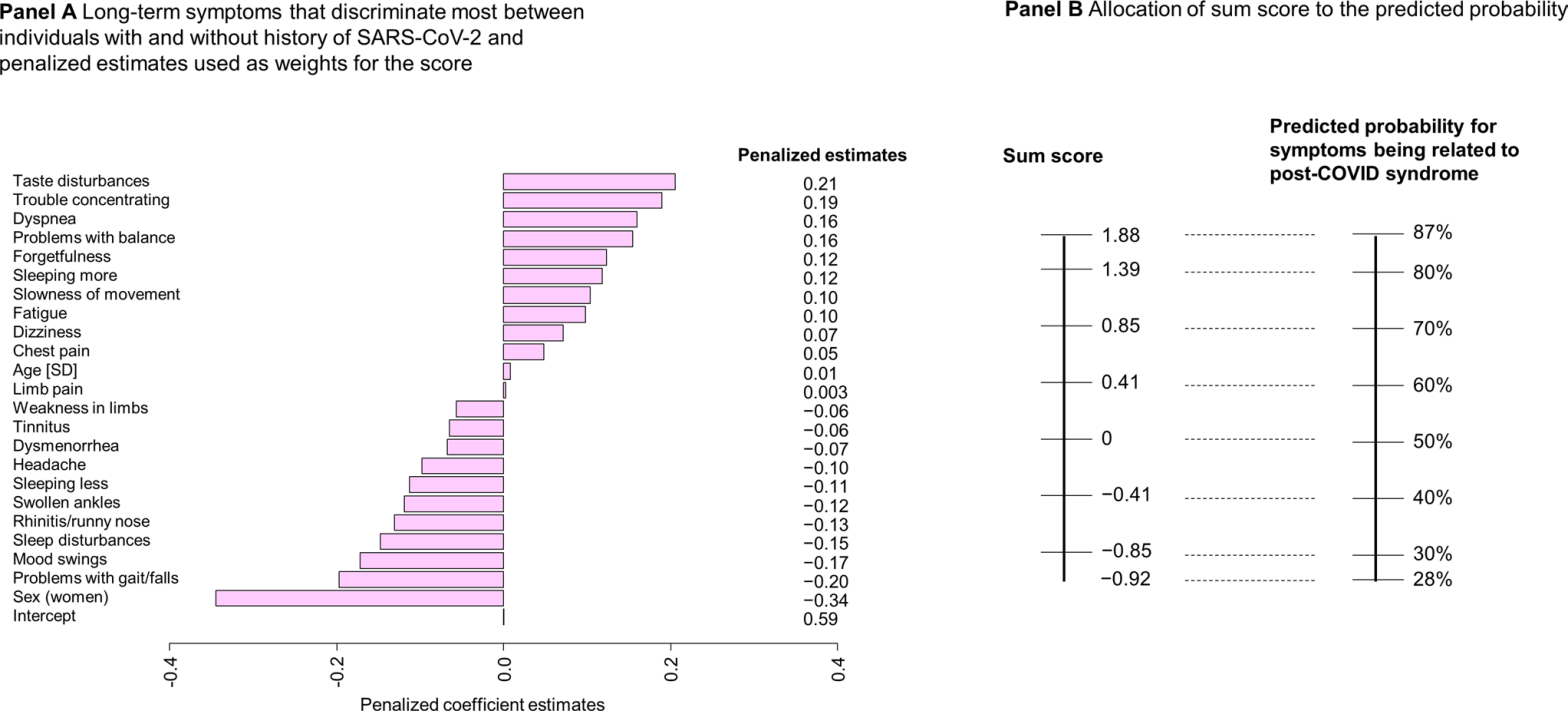
Development of the GCS Post-COVID Diagnostic Score. Panel A shows the penalized estimates of the selected symptoms with regularized regression (adjustment for sex and age, AUC = 0.72, 10-fold cross-validation AUC = 0.66, minimal lambda = 0.059, *N* = 652, number of symptoms = 39, selected symptoms = 21). Panel B presents the allocation of the sum score to the predicted probability

The development of the GCS Post-COVID Diagnostic Score is displayed in Fig. 6. In addition to age and sex, 21 symptoms were selected by machine learning that most discriminated between individuals with and without a history of SARS-CoV-2 (Fig. 6, **Panel A**). Long-term taste disturbances were the strongest symptom favoring individuals with a history of SARS-CoV-2 infection followed by trouble concentrating and dyspnea. The corresponding sum score and predicted probabilities are shown in Fig. 6, **Panel B**. The model had an AUC of 0.72 (cross-validated AUC = 0.66), and when fixing the sensitivity to 95%, a specificity of 48% was reached (Supplemental Fig. 3). Considering the NPV, 99% of truly negative individuals could be correctly ruled out of having PCS (PPV = 9%).

### Prospective validation of post-COVID scores in an independent population-based cohort

For the validation of the GCS Post-COVID Risk Score, data was prospectively collected for 6,570 individuals and regarding the GCS Post-COVID Diagnostic Score for 3,176 individuals in SentiSurv RLP. In the total sample (*N* = 17,585), the proportion of women was 54.7%, and participants had a mean age of 51.0 (36.0/62.0) years (Supplemental Table 3). When applying the same cut-offs as in the development cohort, the GCS Post-COVID Risk score had an AUC of 0.72 (sensitivity = 95%, specificity = 18%, Supplemental Table 4), and the GCS Post-COVID Diagnostic Score had an AUC of 0.64 (sensitivity = 51%, specificity = 68%, Supplemental Table 5).

## Discussion

This work investigated the symptom profile of long-term sequelae after known and undetected SARS-CoV-2 infections compared with a SARS-CoV-2-free control group, identified clinical risk factors, and generated two data-driven scores via machine learning techniques to assist primary care physicians in the initial management of potential PCS patients. The results indicate a substantial prevalence of post-COVID-like sequelae among individuals with a known and undetected history of SARS-CoV-2. The reported symptoms were heterogeneous and did not show a clear pattern, which emphasizes that symptoms are non-specific, symptom clusters are only conditionally indicative of a diagnosis, and individuals must be systematically examined in the sense of a diagnosis of exclusion. The low specificity of post-COVID-like symptoms was underscored by the high proportion of individuals without history of SARS-CoV-2 reporting persistent post-COVID-like symptoms, such as fatigue. However, differences in symptomology between groups were identified. Individuals with certain health conditions before infection and specific symptoms during the acute infection were confirmed as having a higher risk for post-COVID-like sequelae.

### Symptom profile of PCS

The heterogeneity of long-term symptoms described in the literature is also evident in this analysis. Still, consistent with several meta-analyses, fatigue was the most commonly reported symptom among individuals with a history of known SARS-CoV-2 infection [2, 4, 14, 25, 26]. However, persistent fatigue has also been reported in persons without SARS-CoV-2, highlighting the need to compare symptoms between individuals with and without a history of SARS-CoV-2 infection. In line with multiple population-based studies from Germany, US, UK, and Israel [27–33], present results indicate that fatigue, dyspnea, anosmia, forgetfulness, trouble concentrating, and problems with balance are associated with PCS. Fatigue and neurocognitive impairment were suggested to have the greatest impact on self-reported health recovery and ability to work [34]. A population-based study revealed that the symptom pattern of PCS is similar across the wild type, alpha, and delta variant for SARS-CoV-2 [32]. However, the prevalence of symptoms decreased over time, which was also observed over a longer period of time [35]. The multi-symptom involvement across several organ systems noted in this work reflects existing literature and thus supports the proposed concept that PCS is a multi-system disease [6, 36]. The identified symptoms characterizing PCS also contribute to the knowledge of the pathophysiology of PCS. Trouble concentrating and forgetfulness, indicating “brain fog”, fatigue, dyspnea, and loss of smell were suggested to be related to viral persistence of SARS-CoV-2, which is currently discussed as a putative pathomechanism of PCS [37]. The disturbances in balance and dyspnea may indicate the involvement of autonomic dysfunction in PCS. In other studies, dizziness, palpitations, chest pain, and changes in sexual desire or capacity were found to be key symptoms of PCS, supporting the involvement of autonomic dysfunction [28, 30, 32, 38]. In contrast, headache, sleep disturbances, sleeping less, and loss of interest or pleasure are more likely attributable to another condition. Symptoms that are more prevalent in persons without a history of SARS-CoV-2 infection may also indicate societal and psychosocial effects of the pandemic itself [9, 28, 39]. This is particularly reflected in the frequent reporting of loss of interest or pleasure since the pandemic in individuals without SARS-CoV-2 infection.

### Scores for risk and diagnostic assessment of PCS

Physicians in primary care in particular need easy-to-use tools to decide on further diagnostics. Both developed scores are solely based on self-reported symptoms, the first and often only source of information available to physicians in primary care.

The GCS Post-COVID Risk Score provides a tool for prognosing the risk of long-term sequelae based on symptoms experienced during the acute infection. In contrast to the “PASC score” developed by Cervia et al., no information on the history of asthma bronchiale and blood measurements is needed [40]. It is, therefore, particularly suitable for use by general practitioners. Another prediction model for risk prognosis used the number of symptoms in the first week of infection along with age and sex [41]. However, this score was used to predict PCS 28 days after SARS-CoV-2 infection rather than long-term symptoms. The GCS Post-COVID Risk Score can thus identify risk groups for long-term sequelae in need for preventive measures.

The lack of an established diagnostic tool results in over- or underdiagnosing PCS [9]. The GCS Post-COVID Diagnostic Score provides a probability of the presence of PCS, which is urgently needed in primary care due to the high number of patients with long-term symptoms and the low number of PCS-specific symptoms. Very few available scores can be used in the diagnostic setting of PCS. The “PCS score” developed by Bahmer et al. is used to classify PCS severity [9, 42] and is unsuitable for diagnosing PCS since a control group was lacking. The “PCS score” was successfully applied in a German multi-center study that mainly included individuals hospitalized for acute COVID-19 [42]. Cluster analysis of a multinational cohort of outpatients and inpatients with SARS-CoV-2 revealed four clinical phenotypes of PCS that can be used to define the heterogeneous syndrome [36]. In a multi-center study across the United States, symptoms that discriminate between individuals with and without a history of SARS-CoV-2 were selected via LASSO to identify PCS cases [30]. The index has been recently refined with more recruited participants in the derivation cohort [43]. Similar to the GCS Post-COVID Diagnostic Score, fatigue, brain fog, dizziness, taste disturbances, chest pain, and dyspnea were selected. The index was developed without individuals with a history of undetected SARS-CoV-2 infection and has not been validated in an independent cohort.

### Strengths and limitations

The major strengths in this project include the control group that was proven to be free of SARS-CoV-2 in a multimodal diagnostic approach and the prospective validation of the PCS scores in a large, independent cohort. Another strength is the consideration and investigation of individuals with a history of undetected SARS-CoV-2 infections, who are a highly relevant group given the high number of unreported cases. The generalizability of the tools for the risk and diagnostic assessment of PCS in the population is enhanced by the population-based setting.

Nevertheless, some limitations should be considered. The symptoms during the acute SARS-CoV-2 infection and sequelae after 3 and 6 months were assessed simultaneously and retrospectively, which may have led to a recall bias. Symptoms were self-reported, which could be influenced by various factors, including age, sex, socioeconomic status, and cultural background. For individuals with a history of undetected SARS-CoV-2 infection, symptoms were assessed since the onset of the pandemic. Therefore, the symptoms may not reflect a SARS-CoV-2 infection but another (infectious) disease. Nevertheless, the prevalence of other infections, such as influenza, was at an all-time low during the pandemic, especially during data collection (2020–2021) [44, 45]. Since individuals with a history of undetected SARS-CoV-2 infection and the control group were asked about persistent symptoms since the pandemic, the time periods are longer than those for persons with a history of known SARS-CoV-2 infection who were asked about persistent symptoms since the infection. The data for developing the scores capture time periods when the wild type and the alpha variants of SARS-CoV-2 were dominant. Evidence suggests that there are sub-phenotypes of symptoms for the acute infection with SARS-CoV-2 [46] and PCS [12], depending on the variant. Hence, the utility of the developed scores should be evaluated in the newer virus variants. However, the scores were validated successfully in a cohort at a time when newer variants such as Omicron were present. The predicted probabilities of the scores are constrained within a range of 27–87%. Generally, the performance of the scores depends on the pre-test probability of PCS, which is currently difficult to estimate [4], and SARS-CoV-2 infections, which vary seasonally. When validating both scores, the prevalence was not adapted to maintain the user-friendly format. To account for differences in prevalence in different settings, the scores can be calibrated by adjusting the intercept. Due to these limitations, the scores should be used in conjunction with other clinical markers and medical judgment.

## Conclusion

Individuals with and without SARS-COV-2 infection reported persistent and partly comparable symptoms, however, differences in the symptomology of these groups with a history of SARS-CoV-2 were identified, and a specific symptom profile for PCS was derived. The study underscores PCS as a multi-system disease, potentially involving autonomic dysfunction. The newly developed Post-COVID Risk and Post-COVID Diagnostic Scores provide valuable tools for the clinical management of PCS patients in the primary care setting, relying only on self-reported symptoms.

### Declarations

## Electronic supplementary material

Below is the link to the electronic supplementary material.

Supplementary Material 1
