# Supplementary material for: Risk tools for predicting long-term sequelae based on symptom profiles after known and undetected SARS-CoV-2 infections in the population
Source: Eur J Epidemiol. 2025 May 19;40(7):789–801. doi: 10.1007/s10654-025-01223-y (PMC12304058; doi:10.1007/s10654-025-01223-y)
Supplement: Supplementary file 1 — Supplementary Material 1 [file 10654_2025_1223_MOESM1_ESM.docx]

**SUPPLEMENTARY APPENDIX**

Risk tools for predicting long-term sequelae based on symptom profiles after known and undetected SARS-CoV-2 infections in the population

R. Baumkötter, MSc^1,2^; S. Yilmaz, MSc^1,2^; J. Chalabi, MA^1^; V. ten Cate, PhD^1,2,3^; A. S. Mamoor Alam, MSc^1,2^; S. Golriz Khatami, PhD^1^; D. Zahn, PhD^1,2,4^; N. Hettich-Damm, PhD^5^; J. H. Prochaska, MD^1,2,3^; I. Schmidtmann, PhD^6^; K. Lehnert, MD^7,8^, A. Steinmetz, MD^8,9^, M. Dörr, MD^7,8^, N. Pfeiffer, MD^10^; T. Münzel, MD^2,11^; K. J. Lackner, MD^2,12^; M. E. Beutel, MD^5^; P. S. Wild, MD MSc^1,2,3,13^

1. Preventive Cardiology and Preventive Medicine, Center for Cardiology, University Medical Center of the Johannes Gutenberg University Mainz, Langenbeckstr. 1, 55131 Mainz, Germany;
2. German Center for Cardiovascular Research (DZHK), Partner Site Rhine Main, University Medical Center of the Johannes Gutenberg University Mainz;
3. Center for Thrombosis and Hemostasis (CTH), University Medical Center of the Johannes Gutenberg University Mainz, Langenbeckstr. 1, 55131 Mainz, Germany;
4. Health Sciences, Hochschule Fulda University of Applied Sciences, Leipziger Str. 123, 36037 Fulda, Germany;
5. Department of Psychosomatic Medicine, University Medical Center of the Johannes Gutenberg University Mainz, Untere Zahlbacher Str.8, 55131 Mainz, Germany;
6. Institute of Medical Biometry, Epidemiology, and Informatics, University Medical Center of the Johannes Gutenberg University Mainz, Rhabanusstraße 3, Tower A, 55131 Mainz, Germany;
7. Department of Internal Medicine B, University Medicine Greifswald; Fleischmannstraße 8, 17475 Greifswald;
8. German Center for Cardiovascular Research (DZHK), Partner Site Greifswald;
9. Physical and Rehabilitation Medicine, Department of Orthopaedics,Trauma and Rehabilitation Medicine, University Medicine Greifswald; Ferdinand-Sauerbruch-Straße, 17475 Greifswald;
10. Department of Ophthalmology, University Medical Center of the Johannes Gutenberg University Mainz, Langenbeckstr. 1, 55131 Mainz, Germany;
11. Cardiology I, Center for Cardiology, University Medical Center of the Johannes Gutenberg University Mainz, Langenbeckstr. 1, 55131 Mainz, Germany;
12. Institute of Clinical Chemistry and Laboratory Medicine, University Medical Center of the Johannes Gutenberg University Mainz, Langenbeckstr. 1, 55131 Mainz, Germany;
13. Institute of Molecular Biology (IMB), Ackermannweg 4, 55128 Mainz, Germany.

**Correspondence**

Philipp S. Wild, MD, MSc

Professor of Clinical Epidemiology

University Medical Centre Mainz of the Johannes Gutenberg University Mainz, Langenbeckstr. 1, 55131 Mainz, Germany

Phone: +49 6131 17 7163; Fax: +49 6131 17 8460;

Email: philipp.wild@unimedizin-mainz.de

**TABLE OF CONTENTS**

| Title | Page |
| --- | --- |
|  |  |
| Additional information on PCR and antibody testing | 3 |
|  |  |
| Assessment of acute and long-term symptoms of SARS-CoV-2 infection | 4 |
|  |  |
| Interaction of the ten most common symptoms among individuals with a history of SARS-CoV-2 with age and sex | 6 |
|  |  |
| Prevalence of long-term symptoms among individuals with a history of known SARS-CoV-2 infection compared to those with a history of undetected SARS-CoV-2 infection | 7 |
|  |  |
| ROC-curve, 2x2 contingency table, and performance values of the GCS Post-COVID Risk Score | 8 |
|  |  |
| ROC-curve, 2x2 contingency table, and performance values of the GCS Post-COVID Diagnostic Score | 9 |
|  |  |
| Characteristics of SentiSurv RLP – validation cohort | 10 |
|  |  |
| Validation of the GCS Post-COVID Risk Score | 11 |
|  |  |
| Validation of the GCS Post-COVID Diagnostic Score | 11 |
|  |  |

**Additional information on PCR and antibody testing**

*RT-qPCR Testing*

To detect an acute SARS-CoV-2 infection, a swab was taken from the participant’s throat for quantitative reverse transcription polymerase chain reaction (RT-qPCR) analysis. Samples (swabs in 750 µl phosphate-buffered saline) were collected, stored at 4-8°C, and analyzed within 4 days at the latest. For SARS-CoV-2 screening, 200 µl of swab fluid was pooled from each of 5 subjects, RNA was extracted using the QiaAmp UltraSens Virus Kit (Qiagen) and eluted in 30 µl buffer. 10 µl of the eluate were assayed in a 25 µl one-step reverse transcription / qPCR reaction on a cobas z 480 Analyzer (Roche Deutschland Holding GmbH, Germany). Primer and probes for E-gene (plus EAV control) amplification were supplied by TIB MolBiol (Light Mix SarbecoV, TIB Molbiol, Germany), reagents for reverse transcription and qPCR by Qiagen (One-Step QuantiTect Probe RT-PCR Kit, Qiagen, Germany). Details on assay implementation, quality and quality control are described in Hauser et al. (1, methods: ‘laboratory 2’). In case of a positive pool, all samples were re-tested individually by E-gene and RdRP-gene (Light Mix SarbecoV, TIB Molbiol, Germany) qPCR as described in Hauser et al. (1; methods: ‘laboratory 2’).

*SARS-CoV-2 Antibody Testing*

EDTA samples were analyzed regarding circulating antibodies targeted at SARS-CoV-2 nucleocapsid with a qualitative microparticle chemiluminescent immunoassay (Architect SARS-CoV-2 IgG, Abbott, Germany) with a threshold of 1.4 relative light units and second, a qualitative microparticle electro-chemiluminescence immunoassay (Elecsys Anti-SARS-CoV-2 Pan-Ig, Roche, Germany) with a cutoff index of 0.8. Samples were collected and stored at room temperature and were analyzed with the Abbott immunoassay within two days at the latest. Subsequently, samples were stored at −80°C in a state-of-the-art biobanking facility at the University Medical Center Mainz. After defrosting the samples, they were re-analyzed with the immunoassay of Elecsys Anti-SARS-CoV-2 Pan-Ig (Roche, Germany).

Reference

Hauser F, Sprinzl MF, Dreis KJ, Renzaho A, Youhanen S, Kremer WM, et al. Evaluation of a laboratory-based high-throughput SARS-CoV-2 antigen assay for non-COVID-19 patient screening at hospital admission. Med Microbiol Immunol. 2021;210(2-3):165-71.

**Supplemental Table 1.** Assessment of acute and long-term symptoms of SARS-CoV-2 infection

| ***Example for three symptoms (items translated from German to English)***  *“In the following, I will now ask you questions about possible symptoms of your corona disease at different times after the start of the infection. Did you have any of the following new symptoms or symptoms that were significantly more severe than usual?”* | | |
| --- | --- | --- |
| Sleep disturbances | If yes, then follow-up question | 0= no  1= yes  2= don't know |
|  | At what times did you have the complaints? | 1= 0-3 months after infection  2= 3-6 months after infection  3= 6 months and longer after infection |
|  | How intense did you experience these complaints? | 1= light  2= moderate  3= severe |
|  | Did you already have these symptoms regularly before the infection? | 0= No, they are new  1= Yes, lighter  2= Yes, the same  3= Yes, worse  4= Don't know |
| Sleeping less | If yes, then follow-up question | 0= no  1= yes  2= don't know |
|  | At what times did you have the complaints? | 1= 0-3 months after infection  2= 3-6 months after infection  3= 6 months and longer after infection |
|  | How intense did you experience these complaints? | 1= light  2= moderate  3= severe |
|  | Did you already have these symptoms regularly before the infection? | 0= No, they are new  1= Yes, lighter  2= Yes, the same  3= Yes, worse  4= Don't know |
| Sleeping more | If yes, then follow-up question | 0= no  1= yes  2= don't know |
|  | At what times did you have the complaints? | 1= 0-3 months after infection  2= 3-6 months after infection  3= 6 months and longer after infection |
|  | How intense did you experience these complaints? | 1= light  2= moderate  3= severe |
|  | Did you already have these symptoms regularly before the infection? | 0= No, they are new  1= Yes, lighter  2= Yes, the same  3= Yes, worse  4= Don't know |

Symptoms were assessed in a telephone interview according to according to the WHO Case Report Form for Post-COVID condition (Post COVID-19 CRF).

Reference

World Health Organization (WHO). Global COVID-19 Clinical Platform - Case Report Form (CRF) for Post COVID condition (Post COVID-19 CRF). (2021).

**
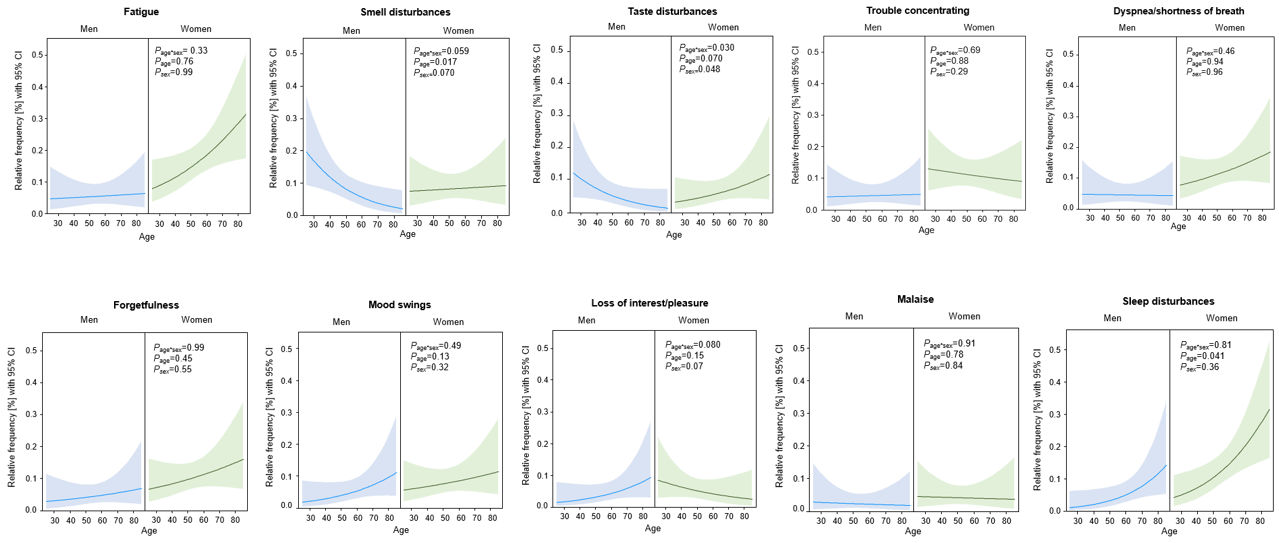
Supplemental Figure 1.** Interaction of the ten most common symptoms among individuals with a history of SARS-CoV-2 with age and sex.

**Supplemental Table 2.** Prevalence of long-term symptoms among individuals with a history of known SARS-CoV-2 infection compared to those with a history of undetected SARS-CoV-2 infection.

|  | **History of known SARS-CoV-2 infection vs. history of undetected SARS-CoV-2 infection** | |  |
| --- | --- | --- | --- |
|  | Prevalence ratio  [95% CI] | *P* | Missing  % |
|  |  |  |  |
| Any long-term symptom | **1.57 [1.17; 2.10]** | **0.0025** |  |
|  |  |  |  |
| *Specific long-term symptoms* |  |  |  |
| Anxiety | 1.86 [0.44; 7.77] | 0.40 | 3.9% |
| Behavior change | 0.88 [0.22; 3.46] | 0.85 | 3.9% |
| Chest pain | 1.53 [0.40; 5.88] | 0.53 | 3.4% |
| Constipation | 0.25 [0.05; 1.19] | 0.081 | 4.1% |
| Depression | 2.51 [0.79; 7.95] | 0.12 | 4.4% |
| Diarrhea | 0.62 [0.07; 5.57] | 0.67 | 3.7% |
| Dizziness | 1.63 [0.55; 4.78] | 0.38 | 4.1% |
| (Dry) cough | 1.56 [0.48; 5.06] | 0.46 | 3.9% |
| Dysmenorrhea | **0.22 [0.06; 0.82]** | **0.025** | 10.8% |
| Dyspnea | 1.03 [0.52; 2.03] | 0.93 | 3.7% |
| Erectile dysfunction | 1.90 [0.34; 10.71] | 0.47 | 12.4% |
| Fatigue | **2.14 [1.15; 4.00]** | **0.017** | 5.3% |
| Forgetfulness | 2.10 [0.97; 4.54] | 0.060 | 5.7% |
| Hair loss | 2.71 [0.86; 8.61] | 0.090 | 3.4% |
| Headache | 0.60 [0.21; 1.72] | 0.35 | 4.1% |
| Jerking of limbs | 1.10 [0.09; 12.81] | 0.94 | 4.1% |
| Joint pain/swelling | 1.06 [0.49; 2.31] | 0.88 | 3.9% |
| Limb pain | 1.27 [0.37; 4.36] | 0.70 | 4.4% |
| Loss of interest/pleasure | 1.98 [0.71; 5.47] | 0.19 | 4.6% |
| Malaise | 2.10 [0.60; 7.31] | 0.25 | 4.4% |
| Mood swings | 1.04 [0.49; 2.21] | 0.92 | 6.0% |
| Muscle pain | 1.86 [0.61; 5.67] | 0.28 | 4.1% |
| Numbness or tingling | 0.80 [0.20; 3.11] | 0.74 | 4.6% |
| Palpitations | 0.86 [0.33; 2.30] | 0.77 | 4.8% |
| Problems hearing | 3.10 [0.86; 11.23] | 0.084 | 3.7% |
| Problems passing urine | 1.19 [0.15; 9.36] | 0.87 | 3.7% |
| Problems seeing | 1.31 [0.32; 5.31] | 0.71 | 3.7% |
| Problems with balance | 1.83 [0.70; 4.78] | 0.22 | 4.4% |
| Problems with gait/falls | 1.16 [0.32; 4.21] | 0.82 | 3.9% |
| Rhinitis/runny nose | 0.40 [0.06; 2.64] | 0.34 | 4.6% |
| Seizures | 5.22 [0.92; 29.79] | 0.063 | 3.7% |
| Sleep disturbances | 1.20 [0.62; 2.35] | 0.59 | 4.8% |
| Sleeping less | 1.30 [0.46; 3.71] | 0.62 | 4.1% |
| Slowness of movement | 0.89 [0.20; 4.00] | 0.88 | 4.4% |
| Smell disturbances | **4.95 [1.81; 13.59]** | **0.0019** | 4.1% |
| Sore throat/throat scratching | 1.48 [0.30; 7.22] | 0.63 | 5.1% |
| Swollen ankles | 1.15 [0.29; 4.63] | 0.84 | 3.9% |
| Taste disturbances | **8.41 [2.08; 34.08]** | **0.0029** | 3.7% |
| Tinnitus/ringing in ears | 1.24 [0.40; 3.84] | 0.71 | 3.7% |
| Tremor | 1.78 [0.36; 8.71] | 0.48 | 3.9% |
| Trouble concentrating | **2.40 [1.09; 5.29]** | **0.030** | 4.4% |
| Weakness in limbs | 2.09 [0.45; 9.82] | 0.35 | 3.7% |
|  |  |  |  |

**Supplemental Figure 2.** ROC-curve, 2x2 contingency table, and performance values of the GCS Post-COVID Risk Score**.**

**Panel A.** ROC-curve.

**
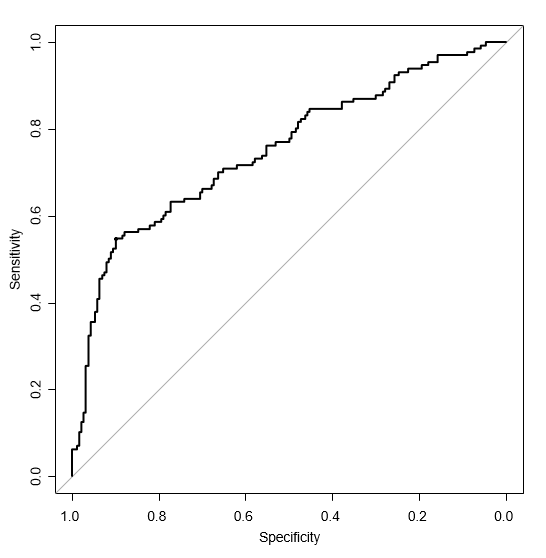
**

**Panel B.** 2x2 contingency table.

|  | Observed  sequelae | Observed  non-sequelae | **Total** |
| --- | --- | --- | --- |
| Predicted sequelae | 124 | 159 | 283 |
| Predicted non-sequelae | 15 | 77 | 92 |
| **Total** | 139 | 236 | 375 |

**Panel C.** Performance of the score.

| **Performance values** | **%** |
| --- | --- |
|  |  |
| Sensitivity | 93.5 |
| Specificity | 19.9 |
| Positive predicted value* | 35.5 |
| Negative predicted value* | 86.7 |
| Accuracy* | 43.5 |
|  |  |

*Dependent on an 32% prevalence of long-term sequelae (new onset or worsened symptom persistent for at least 6 months after SARS-CoV-2 infection).

**Supplemental Figure 3.** ROC-curve, 2x2 contingency table, and performance values of the GCS Post-COVID Diagnostic Score.

**Panel A.** ROC-curve.


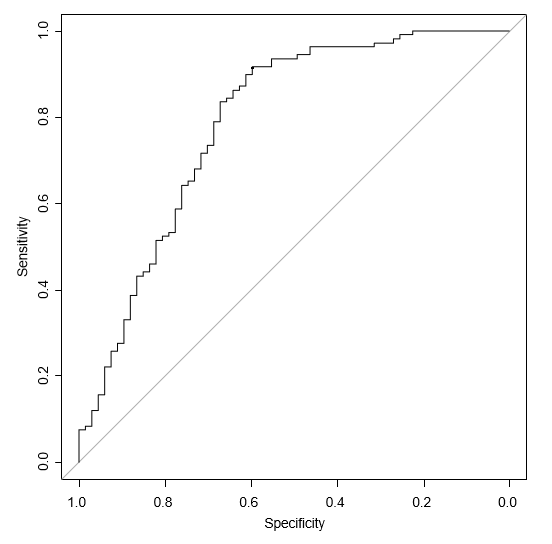


**Panel B.** 2x2 contingency table.

|  | Observed  infected | Observed  non-infected | **Total** |
| --- | --- | --- | --- |
| Predicted infected | 103 | 35 | 138 |
| Predicted non-infected | 6 | 32 | 38 |
| **Total** | 109 | 67 | 176 |

**Panel C.** Performance of the score.

| **Performance values** | **%** |
| --- | --- |
|  |  |
| Sensitivity | 94.5 |
| Specificity | 47.8 |
| Positive predicted value* | 8.7 |
| Negative predicted value* | 99.4 |
| Accuracy* | 50.1 |
|  |  |

*Dependent on an 5% prevalence of SARS-CoV-2 infections.

**Supplemental Table 3.** Characteristics of SentiSurv RLP – validation cohort.

|  | **History of**  **SARS-CoV-2 infection**  N=13,130 | **Control group without SARS-CoV-2 infection**  N=4,455 |
| --- | --- | --- |
|  |  |  |
| *Sociodemographic data* |  |  |
| Sex (women) [%] (n) | 55.1 (7,234) | 53.4 (2,379) |
| Age [years] (IQR) | 49.0 (35.0/60.0) | 56.0 (41.0/65.0) |
|  |  |  |
| *Traditional cardiovascular risk factors [%] (n)* | | |
| Arterial hypertension | 24.7 (2,698) | 32.9 (1,208) |
| Diabetes mellitus | 20.4 (2,670) | 23.1 (1,022) |
| Obesity | 16.2 (2,132) | 19.9 (888) |
| Smoking | 11.2 (1,465) | 15.0 (667) |
|  |  |  |
| *Clinical profile [%] (n)* |  |  |
| Autoimmune disease | 9.8 (1,061) | 11.5 (421) |
| Atrial fibrillation | 5.2 (541) | 6.1 (214) |
| COPD | 1.0 (134) | 2.6 (116) |
| Coronary artery disease | 1.9 (200) | 3.3 (115) |
| Heart failure | 2.1 (222) | 3.0 (107) |
| Hx. of cancer | 6.0 (783) | 7.6 (339) |
| Hx. of myocardial infarction | 1.0 (130) | 1.7 (75) |
| Hx. of stroke | 0.9 (124) | 1.5 (67) |
| Hx. of venous thromboembolism | 2.9 (376) | 3.9 (174) |
| Kidney disease | 4.1 (446) | 5.6 (207) |
|  |  |  |
| *SARS-CoV-2 infection severity [%] (n)* |  |  |
| Asymptomatic/mild | 35.9 (1,987) | n.a. |
| Moderate | 45.3 (2,508) | n.a. |
| Severe | 18.9 (1,045) | n.a. |
|  |  |  |
| COVID-19 vaccination [%] (n) | 99.6 (12,471) | 99.4 (4,232) |
|  |  |  |

Presented are medians with interquartile ranges (IQR) or absolute and relative frequencies.

Self-reported information only. Hx, history.

**Supplemental Table 4.** Validation of the GCS Post-COVID Risk Score.

**Panel A.** 2x2 contingency table.

|  | Observed  sequelae | Observed  non-sequelae | **Total** |
| --- | --- | --- | --- |
| Predicted sequelae | 1,438 | 4,152 | 5,590 |
| Predicted non-sequelae | 71 | 909 | 980 |
| **Total** | 1,509 | 5,061 | 6,570 |

**Panel B.** Performance of the score.

| **Performance values** | **%** |
| --- | --- |
|  |  |
| Sensitivity | 95.3 |
| Specificity | 18.0 |
| Positive predicted value* | 25.8 |
| Negative predicted value* | 92.7 |
| Accuracy* | 35.8 |
|  |  |

*Dependent on an 23% prevalence of long-term sequelae.

**Supplemental Table 5.** Validation of the GCS Post-COVID Diagnostic Score.

**Panel A.** 2x2 contingency table.

|  | Observed  infected | Observed  non-infected | **Total** |
| --- | --- | --- | --- |
| Predicted infected | 1,354 | 167 | 1,521 |
| Predicted non-infected | 1,308 | 347 | 1,655 |
| **Total** | 2,662 | 514 | 3,176 |

**Panel B.** Performance of the score.

| **Performance values** | **%** |
| --- | --- |
|  |  |
| Sensitivity | 50.9 |
| Specificity | 67.5 |
| Positive predicted value* | 89.2 |
| Negative predicted value* | 20.7 |
| Accuracy* | 53.5 |
|  |  |

*Dependent on an 84% prevalence of SARS-CoV-2 infections.
